# Retraction Note: Flavocoxid, a dual inhibitor of COX-2 and 5-LOX of natural origin, attenuates the inflammatory response and protects mice from sepsis

**DOI:** 10.1186/s13054-023-04440-7

**Published:** 2023-04-18

**Authors:** Alessandra Bitto, Letteria Minutoli, Antonio David, Natasha Irrera, Mariagrazia Rinaldi, Francesco S. Venuti, Francesco Squadrito, Domenica Altavilla

**Affiliations:** 1 Department of Clinical and Experimental Medicine and Pharmacology, Section of Pharmacology, University of Messina, Via C. Valeria Gazzi, 98125 Messina, Italy; 2 Department of Neurosciences, Psychiatry and Anaesthesiology, University of Messina, Via C. Valeria Gazzi, 98125 Messina, Italy

**Retraction Note: Critical Care 2012, 16:R32** 10.1186/1364-8535-16-R32

The Editor in Chief retracted this article because of significant concerns regarding the Western blots in Figs. 2A–F and 3A–D which question the integrity of the data. The authors stated that some of these figures were created by cutting and splicing blots to show representative samples. However, the authors were unable to supply raw images or explain the similarity in appearance of the figures. The Editor-in-Chief therefore no longer has confidence in the integrity of the data in this article. Alessandra Bitto and Francesco Squadrito do not agree to this retraction. Letteria Minutoli, Antonio David and Natasha Irrera had not responded to correspondence from the editor about this retraction. The editor was not able to obtain a current email address for Mariagrazia Rinaldi and Francesco S. Venuti. Allessandra Bitto has stated that Domenica Altavilla is now deceased.

